# A Case of True Hermaphroditism Presenting as a Testicular Tumour

**DOI:** 10.1155/2015/598138

**Published:** 2015-02-03

**Authors:** Michelle Ceci, Edward Calleja, Edith Said, Noel Gatt

**Affiliations:** ^1^Department of Pathology, Mater Dei Hospital, Tal-Qroqq MSD 2090, Malta; ^2^Department of Urology, Mater Dei Hospital, Tal-Qroqq MSD 2090, Malta; ^3^Department of Genetics, Mater Dei Hospital, Tal-Qroqq MSD 2090, Malta

## Abstract

True hermaphroditism represents only 5% cases of all of disorders of sexual differentiation (DSD) and usually present in early childhood with ambiguous genitalia. Occasionally, cases might present later on in adolescence with problems of sexual maturation. Our case report presents a true hermaphrodite with normal male phenotype that presented as a left testicular mass, two years after being diagnosed with Sertoli cell only syndrome in the contralateral testis. Histological examination of the left testis showed ovarian, fallopian tube, myometrial, endometrial, and epididymal tissue. This combination of findings is found in approximately one-third of true hermaphrodites, but it is very rare to present clinically as an inguinoscrotal mass.

## 1. Introduction

The term disorder of sexual differentiation (DSD) refers to a child born without clear male or female phenotype [1]. The Metamorphosis, a poem written from the Roman poem Ovid, gives the first account of the term hermaphrodite. True bisexuality that is the ability to function as both a male and female to the point of self-reproduction has never been reported. On the other hand cases of intersexuality (androgyny), there being no complete male and no complete female or both sexes having certain features and organs belonging to each other, have been recognized. True hermaphroditism represents only 5% cases of all of disorders of sexual differentiation (DSD), making it one of the rarest varieties [1]. True hermaphrodites are geographically more prevalent in Africa [2]. The gonads in a true hermaphrodite are asymmetrical having both ovarian and testicular differentiation on either side separately or combined as an ovotestis. In an ovotestis, the testis is always central and ovary polar in location [1].

True hermaphrodites usually present in early childhood with ambiguous genitalia, although presentation in early adulthood is not rare. Late diagnosis of true hermaphrodites may have a severe psychological impact on the patient, hence making early detection and treatment essential [3]. Our case involves a 22-year-old gentleman who presented with testicular pain and a suspicious gonad on the left side.

## 2. Case Study

**Figure 1:**
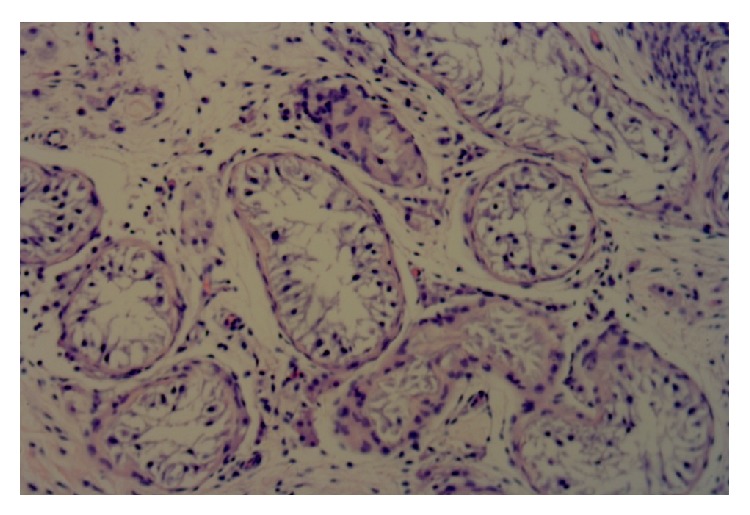
Right (contralateral) testis with seminiferous tubules showing Sertoli cell only syndrome (Magnification ×400).

A 22-year-old gentleman presented in 2012 with right testicular pain. On examination the patient was a phenotypically developed male with normal external male genitalia. His past medical history included recurrent left hypospadias and bilateral mammoplasty. He underwent bilateral testicular exploration during which the right testicle was found to have abnormal features and was biopsied. Histologically there was no malignancy but the seminiferous tubules showed Sertoli cell only syndrome (Figure 1).

Two years later the patient presented with a 2-day history of left dull hemiscrotal ache. On examination a rubbery hard large left testicle was palpated. In view of suspected neoplastic growth an ultrasound was done. This showed an atrophic right testicle and an avascular left testicle. The ultrasound was repeated and confirmed a necrotic left testicle. In view of these findings and the probability of malignancy the patient underwent radical left orchidectomy.

**Figure 2:**
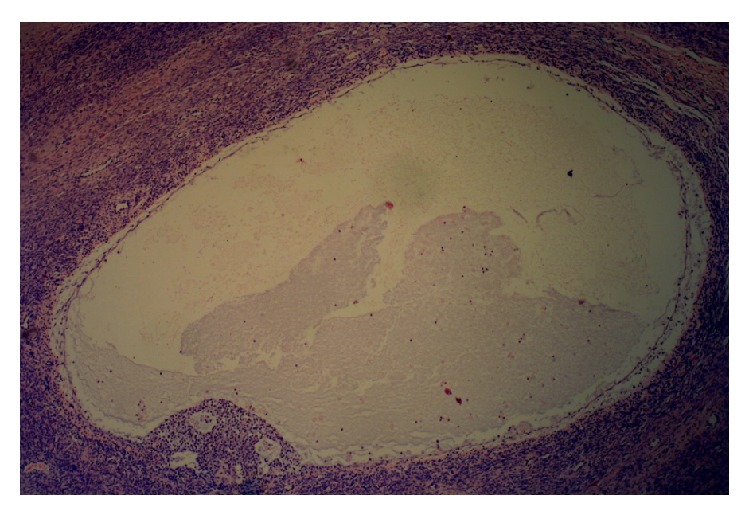
Secondary follicle from the ovarian part of the gonad (Magnification ×100).

**Figure 3:**
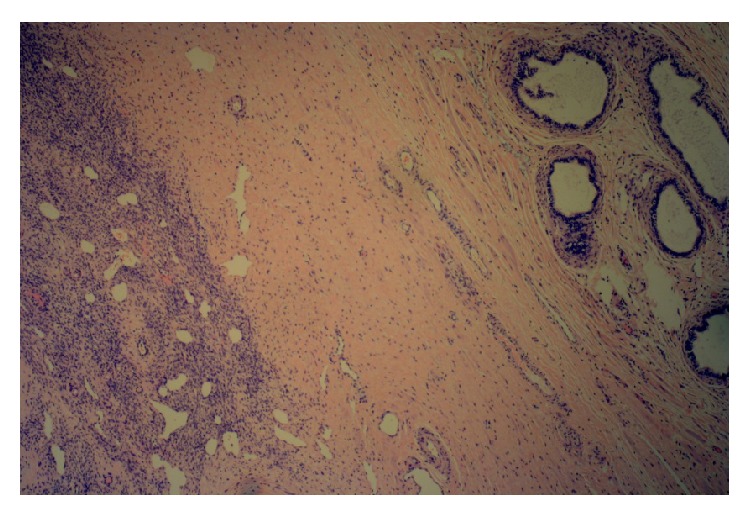
Rudimentary epididymis and ovarian stroma within the right gonad (Magnification ×40).

**Figure 4 fig4:**
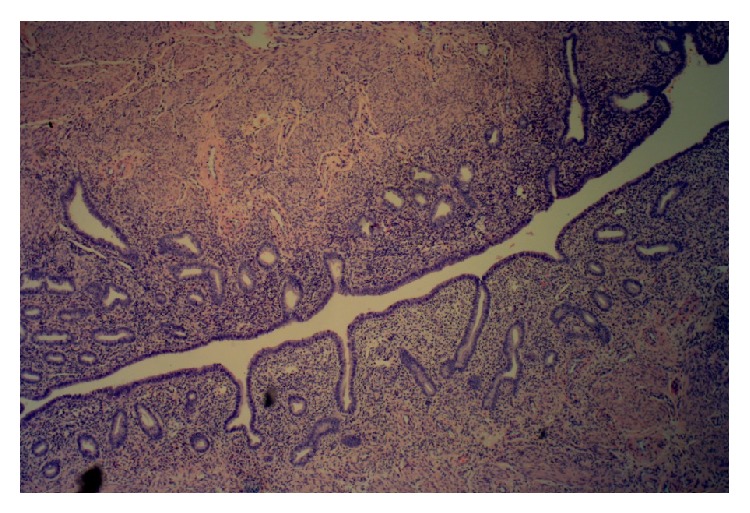
Endometrium and myometrium from the uterine part of the right gonad (Magnification ×40).

**Figure 5:**
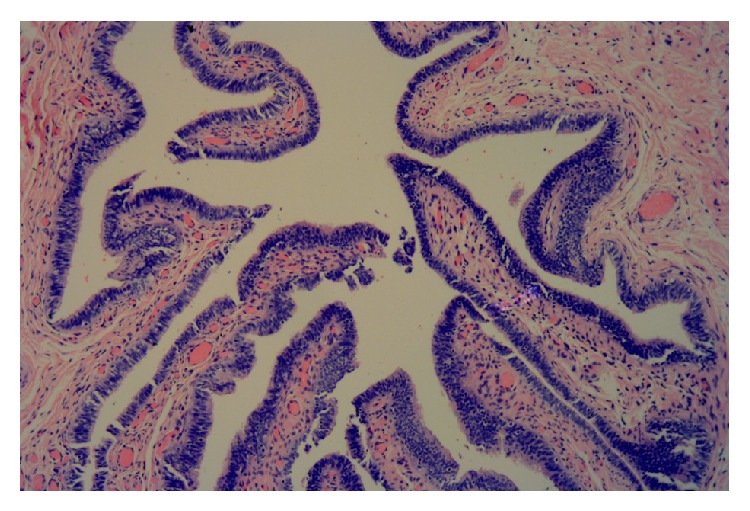
Fallopian tube (Magnification ×100).

The histology of the left gonad showed proper cycling ovarian parenchymal tissue with primary and secondary follicles, a corpus luteum, and several corpora albicantia. Other sections showed mature fallopian tube tissue. A second solid nodular mass attached to the gonad was in fact a functional small uterus (Figures 2 [Fig fig4]–5). A rudimentary epididymis was noted (Figure 3), but seminiferous tubules were not present in this gonad.

A karyotype was conducted and this revealed 46 XX/XY chimera with 11% of the population of cells being XX and 89% of the cell population being XY. Hormonal tests revealed an FSH level of 26.3 U/L (0.711.1 U/L), LH level of 9.8 U/L (0.8–7.6 U/L), testosterone level of 15.50 nmol/L (5.55–25.2 nmol/L), and an oestradiol level of 135 pmol/L (0–206 pmol/L).

## 3. Discussion

The diagnosis of a true hermaphrodite depends on the histologic confirmation of testicular and ovarian tissue in the same individual [1, 4]. 90% of cases present at birth with ambiguous genitalia including microphallus, hypospadias, urogenital sinus, fusion of penoscrotal labia, or cryptorchidism [2]. Individuals which present with even the slightest of ambiguity in the external genitalia and unilateral or bilateral undescended testicles should be investigated [4]. Patients may present during their adolescence or adulthood with gynaecomastia, cyclical groin/scrotal pain and haematuria, primary amenorrhea, and infertility [4].

The most common gonad variant found in a true hermaphrodite is an ovotestis, with 50% being found in ovarian position on the right hand side. Ovaries are present in 33% of cases while testicles are found in 22% [4]. The most common combination is ovotestis-ovary, followed by bilateral ovotestis [2]. True hermaphrodites can be classified according to the position and the histology of the gonads [1].

- Lateral: testis and a contralateral ovary (30% of cases).
- Bilateral: both testicular and ovarian tissue, usually represented by an ovotestis that is identified on both sides (50% of cases).
- Unilateral: ovotestis on one side and a testis or ovary on the other side (20% of cases) [1].

The descent and position of the gonad depend on the amount of testicular tissue present [5]. 50% of the ovotestes are found in an abdominal position, while 25% are positioned in the inguinal region and the other 25% are labioscrotal in position. 85% of ovaries are found in the abdomen and 50% of the testes are labioscrotal [5]. The type of internal genitalia found depends on the nature of the adjacent gonad.

A vas deferens and epididymis are formed beside a testicle, while a fallopian tube accompanies an ovary in the majority of cases. Ductal development is variable in the case of an ovotestis. 65% of the ovotestes are accompanied by a fallopian tube while a vas deferens accompanies the rest. The development of a uterus can occur in the presence of an ovotestis/ovary combination [2].

70% of cases of true hermaphrodites have 46 XX karyotype [2]. Only 10% of cases are 46 XY karyotype, while the rest represent complex karyotypes of which the most common is 46 XX/46XY [2, 5]. The presence of a Y chromosome is more often observed in the absence of an ovotestis [5]. The gene YpqA1 determines testicular development. Not all cases of ovotesticular disorder with testicular tissue have this gene. It is thought that this gene is probably translocated on the X chromosome or any other autosomal site in these patients [2]. In some of these patients gonadal mosaicism may be an explanation [5].

The risk of malignancy ranges from 2.6% to 4.6%, although in true hermaphrodites it is lower than in other types of DSD [2]. Since the chance of malignancy is low, prophylactic removal of the gonad is not indicated [1]. The most common neoplasm is a Germ cell tumour, with dysgerminoma being the most common histological type [2].

Imaging modalities that may aid in the diagnosis of true hermaphrodites include ultrasound, MRI, and genitography [6]. Ultrasonic textural differences between testis and ovaries are well recognized. Undescended gonads may pose a challenge to the ultrasonographer because of the small size and echographic pattern that is similar to the adjacent tissues [4]. MR imaging helps to characterize the abnormal pelvic anatomy. These investigations offer excellent soft tissue contrast with no exposure to radiation. However both testes and noncystic immature ovaries have similar signal intensity on T1- and T2-weighted images [6].

Ultrasound is a noninvasive, cheap procedure that should be used as initial screening for assessment of developmental sexual disorders, especially when different approaches (transabdominal, endoluminal, and transperineal) are used [7]. MRI examination should be reserved for cases in which DSD is suspected but ultrasound failed to identify the gonads, or when proper differentiation between clitoral hypertrophy and micropenis is required for proper precorrective surgery assessment [7].

Laparoscopy is also helpful in diagnosing patients with DSD and in the planning of corrective surgery. Biopsy and gonadectomy may also be performed with this technique [2].

Diagnosis of DSD in a patient should ideally be made before the age of 2 years, since psychological problems are more likely after this age [8]. The aim is to choose the gender that carries the best prognosis for reproductive and sexual function and for which the physical appearance and genitalia may be made to look most normal [9]. Thus this may differ from both the genetic sex and the dominant gonad [10]. Female rearing is advised if the phallus is less than 2 cm stretched length or is there is a large vagina in a normal term neonate [8].

Treatment in cases of DSD may involve medical treatment, surgical correction of ambiguous genitalia and removal of dysgenic gonads or mullerian components, and psychological counselling, especially in patients who have presented during their adolescence or adulthood [3]. If surgery needs to be performed ideally, this is done not later than 24 months of age [9].
